# Retrospective Case Series Comparing the Efficacy of Thoracic Epidural With Continuous Paravertebral and Erector Spinae Plane Blocks for Postoperative Analgesia After Thoracic Surgery

**DOI:** 10.7759/cureus.18533

**Published:** 2021-10-06

**Authors:** Promil Kukreja, Timothy J Herberg, Brittany M Johnson, Alexander M Kofskey, Roland T Short, Lisa MacBeth, Christopher Paul, Hari Kalagara

**Affiliations:** 1 Department of Anesthesiology and Perioperative Medicine, University of Alabama at Birmingham, Birmingham, USA; 2 Medicine, University of Alabama at Birmingham School of Medicine, Birmingham, USA; 3 Department of Anesthesiology and Perioperative Medicine, Mayo Clinic, Jacksonville, USA

**Keywords:** thoracotomy, regional anesthesia, pain management, oral morphine equivalents, postoperative analgesia

## Abstract

Perioperative pain management for thoracic surgery plays a vital role in recovery and improved outcomes. In this retrospective study we compare three different regional anesthesia techniques utilized at one institute to provide postoperative analgesia for thoracic surgery. Continuous thoracic epidural analgesia (TEA), thoracic paravertebral block (PVB) and erector spinae plane (ESP) block are compared for postoperative pain management, opioid requirements, postoperative nausea and vomiting (PONV), respiratory events and length of stay. In this study, pairwise comparisons were also performed among the regional techniques with respect to mentioned outcomes.

## Introduction

Thoracic surgery, especially procedures involving thoracotomy, is associated with severe postoperative pain [1,2]. The severity of thoracotomy-associated pain can be attributed to the significant trauma caused when the incision is made, including dislocation of costovertebral joints, retraction of tissue bordering the surgical field, fracture of ribs, injury to intercostal nerves, pleural irritation, and resection of multiple layers of muscle [3]. Pain control following thoracic surgery is vital as uncontrolled pain can lead to respiratory complications such as atelectasis, diaphragmatic dysfunction, respiratory failure, splinting, and ineffective cough resulting in pneumonia [4]. In addition to acute postoperative pain, chronic pain after thoracotomy affects approximately 50% of patients at three and six months after surgery with some studies linking chronic postoperative pain to inadequate pain control immediately following surgery [5]. Pain management for thoracotomy patients is often multimodal, involving pre- and perioperative regional anesthesia as well as non-steroidal anti-inflammatory drugs and opioids throughout the patient’s procedure and recovery process [4].

In terms of regional anesthesia, thoracic epidural analgesia (TEA) is widely considered the gold standard of post-thoracotomy pain management due to its ability to provide effective analgesia in most patients and decrease postoperative pulmonary morbidity [1,4,6]. TEA blocks spinal nerves bilaterally, unlike other commonly used methods of regional anesthesia for thoracic cases [1]. While TEA has been widely studied and has demonstrated a high degree of efficacy in published literature, it’s associated with several adverse effects like the risk of dural puncture, spinal cord injury, epidural hematoma, epidural abscess, hypotension, and urinary retention [1,4,7].

Paravertebral block (PVB) is another anesthetic intervention commonly used to reduce post-operative pain in thoracic surgery patients. While TEA targets nerves within the spinal canal, PVB anesthetizes nerves after they exit the spinal cord and achieves a unilateral block [8]. PVB can be administered as a single bolus, or as a continuous infusion similar to TEA [1]. Risks associated with PVB include hypotension, urinary retention, hematoma, inadvertent intrathecal or epidural spread of the drug, and pneumothorax [8]. The unilateral nature of PVB results in preservation of sympathetic and respiratory function on the side of the body contralateral to the injection, which may account for the reduced frequency of hypotension and urinary retention associated with PVB as compared to TEA [9]. Evidence suggests PVB affects blood pressure and heart rate less significantly than other forms of regional anesthesia and is therefore the intervention of choice in patients with a history of cardiac disease [8,9].

In addition to TEA and PVB, the erector spinae plane (ESP) block is a more recently introduced method of pain management in thoracic surgical cases [10]. The ESP block is an ultrasound-guided paraspinal fascial plane block, where local anesthetic is injected at fascial plane between ESP muscle and its attachment to specific transverse process based on the desired spread [1,11]. Following injection, injected local anesthetic (LA) spreads in both cephalad and caudad direction from point of injection [1]. Because the ESP injection site avoids pleura, major blood vessels, and the spinal cord, complications involving ESP blocks are rare [1,11]. The ESP block has gained popularity due to its simple procedure, high degree of efficacy, excellent safety profile, and wide range of clinical applications across surgical specialties [10,11].

Various published studies have compared the analgesic effects of TEA and PVB in thoracic surgery patients. Many studies found no significant difference in pain relief provided between the two methods [2,7,8]. However, a retrospective case study published in 2021 by Liang and colleagues found that TEA provided statistically significant reduction of pain scores and amount of opioids used in the first 24 hours post-surgery when compared to PVB [9]. These findings are further supported by a prospective study published in 2020 by Yeap and colleagues which found TEA to be superior to PVB in patient pain control and reduction of opioid use over the first 72 hours following video-assisted thoracoscopic surgery [12]. Several studies reported a lower incidence of adverse effects associated with PVB when compared to TEA, especially in the case of urinary retention and hypotension [7,8]. PVB is also associated with lower failure rates than TEA, as well as better postoperative pulmonary function and fewer pulmonary complications [8,9].

Because the ESP block was first described less than a decade ago and is considered a relatively new technique, there are a limited number of published studies comparing ESP to alternative anesthesia techniques in thoracic surgery such as PVB. However, studies published across other surgical fields suggest that ESP and PVB provide a similar, adequate level of postoperative analgesia [10,13]. A randomized control trial performed in 2019 by El Ghamry and colleagues found no significant difference between pain scores, incidence of nausea, and incidence of vomiting in patients receiving an ESP block versus PVB [13]. While studies suggest that the ESP block and PVB display similar levels of analgesic efficacy, the ESP block is generally considered to be safer and easier to perform than PVB in the thoracic region due to superficial needle placement at transverse process, which avoids pleura and major vasculature [11].

While there is an overall lack of randomized controlled trials comparing the efficacy of ESP block to TEA in thoracic surgery specifically, the studies currently published across other surgical specialties support the use of ESP block as a safe, easy to perform, and non-inferior alternative to TEA [14,15]. Compared to TEA, the ESP block has been shown to deliver a similar, adequate level of pain control in the first 48 hours following cardiac surgery [15].

The comparison of TEA, PVB, and ESP blocks together is currently lacking in the anesthesia literature. This retrospective study compared the efficacy of three different regional anesthesia techniques used for postoperative analgesia in patients undergoing thoracic surgery. In this retrospective study, the authors collected data from a single tertiary care institute and intend to study the efficacy of three commonly used regional anesthesia techniques to provide postoperative analgesia after thoracic surgery. They also intended to use the results to judiciously select the type of regional anesthesia method based on type of surgery, safety of technique, patient co-morbidities and associated complications.

## Materials and methods

**Figure 1:**
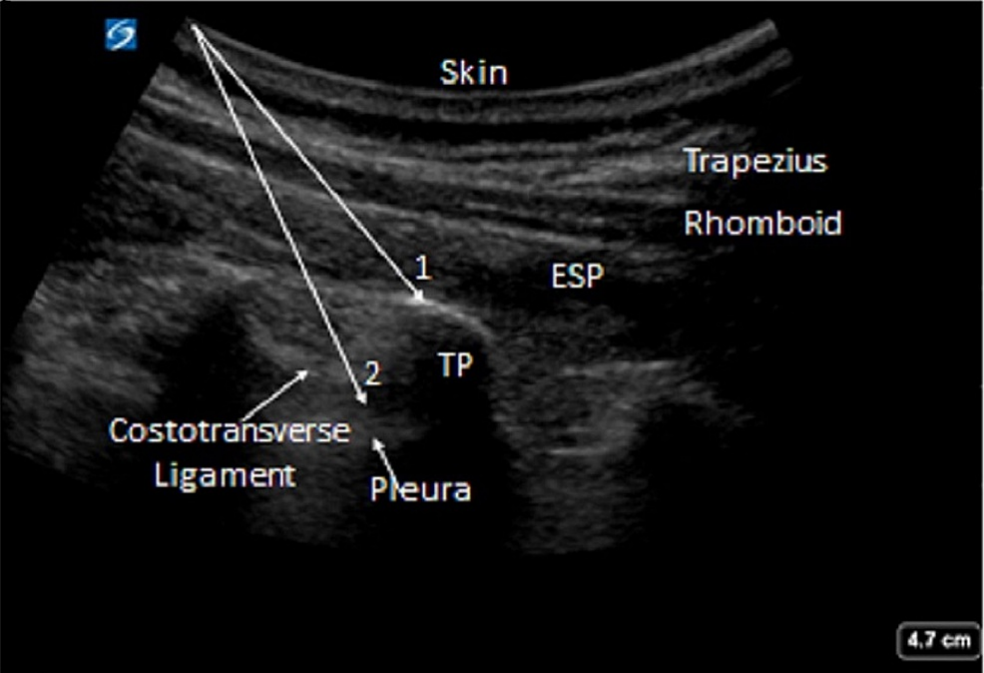
Ultrasound guided ESP (1) and PVB (2) blocks (arrows depict in-plane needle trajectory) ESP: erector spinae plane, PVB: paravertebral block

The patient population for this retrospective cohort study was obtained by searching the acute pain service records at a tertiary care center from July 2019 through November 2020. The Institutional Review Board of University of Alabama at Birmingham issued approval 300006659. Patients were selected if they underwent thoracic surgery (intrathoracic or thoracic wall surgery) and had a preoperative continuous nerve block catheter placed of the following types: erector spinae plane (n=20), paravertebral (n=34), or thoracic epidural (n=96, only first 50 included for data analysis). The majority of the surgeries consisted of video-assisted thoracoscopic or thoracotomies for wedge resection or lobectomy, Ivor Lewis esophagectomy, and pectus repair. Erector spinae plane block and paravertebral blocks were performed under ultrasound guidance (Figure 1) while thoracic epidural blocks were performed by landmark. All blocks were performed by a regional anesthesia fellow or senior resident under direction supervision of an experienced regional anesthesia faculty member. Primary outcomes included visual analog scale (VAS) of pain and oral morphine equivalents (OMEs) between the groups during the following periods, arrival at post-anesthesia care unit (PACU) to PACU discharge, PACU discharge to six hours post-op, six to 12 hours post-op, and 12-24 hours post-op. Secondary outcomes included postoperative (within 24 hours) naloxone administration, documented hypoxic event (oxygen saturation <90% or any supplemental oxygen greater than 6L/min nasal cannula or documented respiratory distress), postoperative reintubation within 24 hours, total length of stay, postoperative nausea and vomiting (evidenced by antiemetic administration), failed block (evidenced by catheter removal within two days of placement not in the setting of hospital discharge), and patient-controlled analgesia (PCA) pump initiation within 24 hours. 

Data were summarized using means and standard errors (SE) for continuous outcomes or counts and percentages for categorical outcomes. One-way ANOVA and chi-square tests were used to compare the three groups. Normality for continuous outcomes was assessed using probability plots and the Shapiro-Wilk test for normality; for any outcomes where normality could not be reasonably assumed, the Kruskal-Wallis test was used in place of ANOVA. For categorical outcomes, Fisher’s exact test was used when assumptions for the chi-square test were not met.

For any continuous outcomes where the overall test was statistically significant, pairwise comparisons were then conducted using the two-sample t-test (or Wilcoxon rank sum test, as appropriate) or chi-square test. Bonferroni adjustment was used to correct for multiple comparisons. A p-value < 0.05 was considered statistically significant. SAS version 9.4 (SAS Institute Inc., Cary, NC, USA) was used to conduct all statistical analyses.

## Results

The three groups did not significantly differ on pain scores in PACU, in the 0- to six-hour postoperative period, the six- to 12-hour postoperative period, or the 12- to 24-hour postoperative period (Table 1). The three groups significantly differed in OME requirements during each time period. There were also significant differences in rate of PONV and PCA within 24 hours of surgery (p < 0.001, for both) (Table 2).

**Table 1:** Pain scores and OMEs for ESP catheter, paravertebral catheter and thoracic epidural in postoperative period. ESP: erector spinae plane, PACU: post-anesthesia care unit, OME: oral morphine equivalent

Table 1. Pain scores and OMEs by anesthesia type.				
Outcome	ESP Catheter (n = 20)	Paravertebral Catheter (n = 34)	Thoracic Epidural (n = 50)	P*
Pain score, mean (SE)				
PACU	4.26 (0.64)	4.32 (0.41)	3.77 (0.38)	0.571
0 to 6 hours	3.94 (0.69)	3.59 (0.41)	2.77 (0.48)	0.280
6 to 12 hours	2.48 (0.57)	2.31 (0.61)	2.76 (0.58)	0.783
12 to 24 hours	3.38 (0.62)	4.17 (0.80)	6.22 (1.10)	0.066
OME, mean (SE)				
PACU	41.80 (10.81)	84.49 (8.89)	20.38 (3.08)	< 0.001
0 to 6 hours	24.90 (7.74)	33.30 (7.60)	5.07 (2.35)	< 0.001
6 to 12 hours	25.90 (9.25)	38.65 (8.39)	17.58 (14.28)	< 0.001
12 to 24 hours	48.23 (14.08)	49.14 (10.17)	13.30 (4.04)	< 0.001
* p-values from Kruskal-Wallis test.				

**Table 2:** Secondary outcomes compared in ESP catheter, paravertebral catheter and thoracic epidural groups ESP: erector spinae plane, PONV: postoperative nausea & vomiting, PCA: patient-controlled analgesia, CICU: cardiac intensive care unit, SICU: surgical intensive care unit

Outcome	ESP Catheter (n = 20)	Paravertebral Catheter (n = 34)	Thoracic Epidural (n = 50)	P*
Post-op Reintubation, N (%)				0.192
No	19 (95.00%)	34 (100.00%)	50 (100.00%)	
Yes	1 (5.00%)	0 (0.00%)	0 (0.00%)	
PONV, N (%)				< 0.001
No	12 (60.00%)	12 (35.29%)	39 (78.00%)	
Yes	8 (40.00%)	22 (64.71%)	11 (22.00%)	
PCA within 24 hours, N (%)				< 0.001
No	14 (70.00%)	9 (26.47%)	47 (94.00%)	
Yes	6 (30.00%)	25 (73.53%)	3 (6.00%)	
Length of Stay, mean (SE)	7.45 (1.26)	6.24 (0.47)	8.42 (0.93)	0.884
Respiratory Event, N (%)				0.405
No	16 (80.00%)	24 (70.59%)	33 (66.00%)	
Other**	1 (5.00%)	0 (0.00%)	1 (2.00%)	
Yes	3 (15.00%)	10 (29.41%)	16 (32.00%)	
Block Failure, N (%)				0.310
No	19 (95.00%)	32 (94.12%)	42 (84.00%)	
Yes	1 (5.00%)	2 (5.88%)	8 (16.00%)	
* p-values from chi-square test (PONV, PCA), Fisher’s exact test (post-op reintubation, respiratory events, block failure), or Kruskal-Wallis test (length of stay). ** Other respiratory events were “intubated coming into CICU” and “on ventilator in SICU”.				

Bonferroni-adjusted p-values for the post-hoc pairwise comparisons for OME requirements, PONV, and PCA within 24 hours are shown in Table 3. In PACU, patients receiving paravertebral catheter had significantly higher OME requirements than both ESP catheter patients (p = 0.029) and thoracic epidural patients (p < 0.001).

**Table 3:** Bonferroni-adjusted p-values for pairwise comparisons. ESP: erector spinae plane, PACU: post-anesthesia care unit, OME: oral morphine equivalent, PONV: postoperative nausea & vomiting, PCA: patient-controlled analgesia

Time	ESP Catheter vs Paravertebral Catheter	ESP Catheter vs Thoracic Epidural	Paravertebral Catheter vs Thoracic Epidural
OME	0.029	0.328	< 0.001
PACU	0.029	0.328	< 0.001
0 to 6 hours	0.999	0.002	< 0.001
6 to 12 hours	0.999	0.028	< 0.001
12 to 24 hours	0.999	0.003	< 0.001
PONV	0.955	0.999	0.001
PCA within 24 hours	0.038	0.132	< 0.001

**Figure 2:**
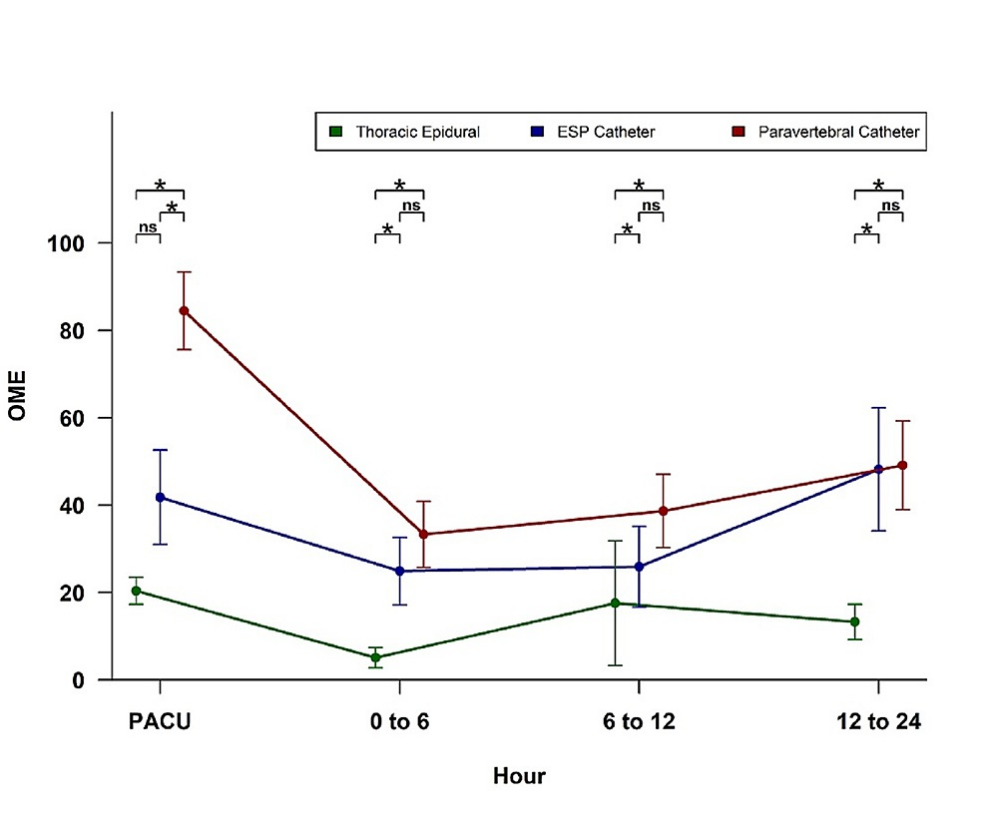
OMEs for thoracic epidural, ESP catheter and paravertebral catheter in postoperative period. ESP: erector spinae plane, PACU: post-anesthesia care unit, OME: oral morphine equivalent

For all remaining time periods, thoracic epidural patients had significantly lower OME requirements than ESP catheter and paravertebral catheter patients; there were no significant differences between ESP catheter and paravertebral catheter patients after PACU (Figure 2). Paravertebral catheter had significantly higher rates of PONV (64.7%) than thoracic epidural (22%); no other pairwise comparisons for PONV were statistically significant after Bonferroni adjustment. Patients receiving paravertebral catheter also had significantly higher probability of PCA within 24 hours of surgery (73.53%) than those receiving either ESP catheter (30%) or thoracic epidural (6%); the ESP catheter and thoracic epidural groups did not significantly differ from each other on this outcome.

## Discussion

There are some established regional anesthesia techniques to provide analgesia for thoracic surgery like thoracic paravertebral block (PVB) and thoracic epidural analgesia (TEA). And there are some novel regional techniques like erector spinae plane (ESP) block, which can be performed at thoracic region to provide analgesia. At our institute we use all three above-mentioned techniques based on procedure, patient co-morbidities, and provider’s preference. In this retrospective study we compared these techniques in relation to postoperative pain scores and OME requirements. We also compared block failure rates, incidence of PONV, re-intubation rates, and length of stay (LOS).

The PVB can be performed both unilaterally (most common) and bilaterally, provided that maximum allowable local anesthetic dose is not exceeded. A Cochrane systematic review concluded that the continuous PVB via catheter was comparable to TEA for thoracotomy [8]. In our study, TEA was superior to PVB and ESP catheter patients with respect to OME requirements and need for PCA within 24 hours in the postoperative period. The low OME requirements correlate well with low incidence of PONV in the TEA group when compared to the PVB group (p< 0.05). Our study results are supported by findings of a recent study comparing PVB and TEA techniques for video-assisted thoracoscopic surgery (VATS) procedure [12]. Improving analgesia and reducing PONV is one of the main goals of enhanced recovery after surgery (ERAS) protocols.

Although block failure rate with TEA was higher (16%) in our study as compared to PVB (~6%), different approaches [16], catheter migration, operator’s skill, and experience may also account for variable success rates. In our study all PVB were performed using ultrasound (US) guidance. The US-guided PVB has less failure rate than the landmark technique [17], which is also supported by our study results where the landmark-based TEA technique has higher percentage failure rates than US-guided PVB. Both catheter techniques are prone to failure secondary to catheter displacement, misplacement, or infection [18]. The short-term postoperative outcomes like post-op re-intubations, respiratory events, and LOS appear to be comparable as quoted in recent studies [19].

The potential for adverse effects (accidental dural puncture, epidural hematoma, hypotension, neurologic injury, etc) from TEA warrants consideration of alternate techniques like PVB or ESP. A meta-analysis of eight prospective randomized controlled studies comparing TEA and PVB for postoperative analgesia in patients undergoing thoracotomy concluded that there was no statistically significant difference in pain scores and PONV between the two techniques, although TEA was associated with higher incidence of hypotension and urinary retention in the postoperative period [7]. Our study did not compare incidence of hypotension and urinary retention in study groups, but there was no reported incidence of dural puncture or epidural hematoma with any technique.

There is limited literature available on the efficacy of ESP block for thoracic surgery barring only a few case reports and prospective studies. The ESP is a relatively new technique where LA is injected in fascial plane between transverse process and erector spinae muscle. The mechanism of action of ESP block is not yet clearly elucidated [20]. Forero et al. [21] described use of ESP block for thoracic neuropathic pain. The authors demonstrated the extent of the cutaneous sensory block from T1 to T11 with cephalocaudad spread of 25 ml of LA injected at the level of T5. They also suggested that likely site of action of ESP block may be dorsal and ventral rami of thoracic spinal nerves.

The ESP block when compared with TEA for postoperative pain management in cardiac surgery revealed comparable VAS scores at 0 h, 3 h 6 h, and 12 h in a recent prospective, randomized comparative clinical study [15]. In this study, incentive spirometry, ventilator, and ICU duration were also comparable. Gurkan et al. [10] reported the efficacy of ESP block as compared to PVB for post-operative analgesia in breast surgery. In this study, opioid consumption was 5.6 ± 3.43 mg in the ESP group, 5.64 ± 4.15 mg in the PVB group, and 14.92 ± 7.44 mg in the control group. In our study, patients receiving PVB catheter had significantly higher OME requirements than both ESP catheter and TEA patients in PACU (p < 0.001). There was no significant difference in OME requirements between ESP catheter and PVB catheter patients after PACU (Figure 1).

The TEA and PVB are mostly chosen for pain management after thoracic surgeries. Intercostal nerve blocks remain as an alternative in case of major contraindication like coagulopathy, or as a rescue in case of failure of these blocks. The ESP block was utilized in this case report [22] as rescue analgesic technique in thoracotomy after failed TEA. Biovicini et al. reported the effective use of bilateral ESP blocks for breast reconstructive surgery and suggested it to be a comparable alternative to PVB and TEA techniques [23].

The clear limitations of this study are its retrospective nature and small number of subjects. Although there was no reported incidence of pneumothorax in our study subjects, the rare adverse events cannot be accurately measured due to small numbers, and also long-term outcomes were not included in this study. Another possible study limitation is variability in operator’s skill level and experience. The blocks were not immediately assessed before surgery to identify failed blocks early on. Sensory testing could be done before surgery to find out the dermatomal distribution and adequacy of block analgesia for particular thoracic surgery.

## Conclusions

Effective perioperative pain management plays a vital role in patients undergoing thoracic surgery. In this study we demonstrated that different regional anesthesia techniques can be utilized to reduce postoperative pain, and opioid consumption. The TEA technique is a superior technique for pain management in thoracic surgeries, but has potential for adverse effects. The ESP block can serve as a useful and safe alternative to either TEA or PVB techniques in thoracic surgeries for perioperative pain management. Future studies are warranted to examine the effect of additives or liposomal bupivacaine to prolong single shot PVB or ESP blocks.
